# Dynamic complexity of Stackelberg-Bertrand game with one-way R&D spillovers, effective information and government subsidies

**DOI:** 10.1371/journal.pone.0328071

**Published:** 2025-07-28

**Authors:** Jianjun Long, Songyin Zheng

**Affiliations:** School of Management Science and Engineering, Chongqing Technology and Business University, Chongqing, China; University of Milano–Bicocca: Universita degli Studi di Milano-Bicocca, ITALY

## Abstract

The phenomena of bounded rationality, asymmetric information, research and development (R&D) spillovers represent ubiquitous characteristics in economic systems, yet research simultaneously investigating R&D spillovers and information asymmetry within a bounded rationality framework remains scarce. This study innovatively incorporates one-way R&D spillovers, effective information, and government R&D subsidies into a dynamical two-stage Stackelberg-Bertrand model. Through application of Jury criterion, we systematically analyze the stability characteristics of all equilibrium points, derive stability conditions and stable regions, and investigate the complex dynamics of this discrete system. The principal findings reveal that: (1) Enhanced effective information for the R&D leader and increased government R&D subsidies exhibit stabilizing effects on equilibrium prices; (2) Excessive product homogeneity may induce substantial price volatility or chaotic dynamics, whereas greater product differentiation enhances profitability for the leader; (3) Moderate levels of R&D spillovers contribute to price stabilization, and while partially reducing leading firms’ profits, they generate positive externalities for enterprise cluster development. This research provides significant theoretical insights and practical implications for innovation strategies and differentiated product development in oligopolistic markets.

## Introduction

Amid the tide of economic globalization, the degree of market openness is continuously increasing, and competition is becoming increasingly fierce. Enterprises are more and more inclined to gain a leading position through innovation. As a major driving force for technological innovation, R&D plays a crucial role in establishing core competitive advantages and enhancing market position for enterprises. Especially in the high-tech field, continuous R&D investment is an effective strategy to ensure that enterprises are not controlled by others and maintain technical independence [1]. “The 2023 European Industrial R&D Investment Scoreboard” has compiled statistics on the top 2500 companies globally in terms of R&D investment. The United States and China, the top two countries in terms of the number of companies, have 827 and 679 companies on the list, respectively, with total R&D investments amounting to 526.5 billion euros and 222 billion euros, accounting for 42.1% and 17.8% of the total R&D investment, respectively. Of the top 20 companies on the list, the United States has 11, including the top four American companies, while China only has Huawei and Tencent entering the list, with Huawei ranking fifth. It is not difficult to see that the R&D intensity of enterprises is closely related to the rise and fall of a country, which has formed a broad consensus. Through high-intensity R&D investment, enterprises can continuously innovate and improve their technical level, thereby promoting the competitiveness of their country in the international market [2].

Despite the widespread acknowledgment among enterprises regarding the crucial role of R&D in fostering long-term growth, a paradoxical trend emerges where these entities exhibit vacillation and risk-averse behavior when contemplating investments in R&D initiatives. This phenomenon is fundamentally rooted in their inherent predilection to avoid high-risk scenarios and their concurrent quest for prompt economic gains, which often supersedes the pursuit of innovative advancements with potentially transformative yet uncertain outcomes. Firstly, R&D activities are inherently a high-cost, high-risk process that requires substantial initial investment, and the outcomes have significant uncertainty [3]. Secondly, firms are concerned that even with substantial R&D investment, they may not be able to achieve corresponding competitive advantages in the market, especially in industries where technology iteration is rapid. Furthermore, the opportunistic behavior caused by R&D spillovers, such as free-riding, suppresses and reduces the enthusiasm of enterprises for R&D [4]. R&D spillovers refer to the phenomenon where knowledge and technological achievements produced by R&D activities are applied and utilized by other enterprises or individuals without consent. Geographical proximity, personnel mobility, commercial espionage, and many other factors lead to the inevitable occurrence of R&D spillovers, especially in clusters where enterprises are concentrated. To stimulate the enthusiasm of enterprises for R&D, reduce their innovation costs and risks, governments provide various effective policy measures such as tax incentives and R&D subsidies to encourage their innovation [5–7]. In this regard, our study will consider both R&D spillovers and government subsidies.

Traditional economics assumes that market participants are completely rational and fully equipped with all the information needed to make decisions, but such assumptions are seriously out of line with social reality. Due to the cognitive limitations of decision makers, the diversity of decision objectives, the differences in information collection and processing capabilities, and the uncertainty of economic environment, it is almost impossible for complete rationality and complete information to exist in the real economy and society. Consequently, the concepts of bounded rationality and information asymmetry have garnered significant scholarly interest. Bounded rational adjustment strategies, such as naïve [8,9], adaptive [4,10], gradient dynamics (GD) [11–13] and local monopolistic approximation (LMA) [14,15] are widely used in monopoly games, while asymmetric information, such as asymmetric cost [4,16], asymmetric access to information [17], and asymmetric market demand [18] have also been discussed in many literatures of bounded rationality. To this end, this paper will take into account the bounded rationality of R&D leader and R&D follower within an enterprise cluster, as well as the asymmetry in their access to information about R&D investment. This will be detailed in the Stackelberg-Bertrand model constructed in Section 3.

Enterprise cluster refers to the phenomenon where a large number of interconnected companies and related support institutions in a particular industry field gather spatially and form a sustainable competitive advantage [19]. It has emerged as a principal model for regional economic development and industrial organization, functioning as an indispensable conduit for stimulating regional economic growth. Owing to geographical proximity, specialized division of labor, and high social trust, the spillover of knowledge and technology distinguishes clustered firms from other general enterprises as a salient characteristic. Research has demonstrated that R&D spillovers constitute a pivotal mechanism underlying the emergence and development of enterprise clusters [20], while also serving as a critical driver enabling clusters to enhance their innovative capacities and secure competitive advantages in the marketplace [21]. Although R&D spillovers would cause enterprises to lose their enthusiasm for R&D due to opportunistic behaviors, and even lead to the similarity and vicious competition of products, the positive externality of R&D spillovers can enhance the social welfare of the whole cluster and improve their technical level [22]. R&D capabilities of cluster enterprises often exhibit a non-uniform distribution, and the judicious selection of technological innovation models is key to enhancing the innovative capacity of the cluster. For instance, leading enterprises typically possess stronger R&D capabilities, enabling them to continuously generate new R&D outcomes, while technologically lagging enterprises have significant differences in R&D levels and knowledge accumulation compared to leading enterprises, making it difficult to form effective reverse spillovers. Similarly, enterprises with rapid technological updates are more likely to become sources of technology spillovers. Therefore, this paper considers a technological innovation model where a leading enterprise engages in independent R&D and a following enterprise imitates R&D, with R&D spillovers flowing unidirectionally from the leader to the follower.

Since it is rare to discuss bounded rationality, asymmetric information and R&D spillover simultaneously in the monopoly games within cluster enterprises, this prompts us to further explore the following questions:(i) The Stackelberg-Bertrand game with bounded rationality and incomplete information is worthy of further analysis. Most literatures analyze the Stackelberg-Cournot model, but ignores the fact of product diversification. Many products are designed to meet the personalized and diversified needs of consumers, so in reality, enterprises often compete on price. (ii) Enterprise cluster is an important economic carrier in today’s world, but R&D games among cluster enterprises have not received much attention under the framework of bounded rationality. (iii) In the oligopoly game between cluster enterprises with bounded rationality and incomplete information, what are the factors that affect the Nash equilibrium, and how do they affect it? To address these issues, we construct a two-stage Stackelberg-Bertrand model including one-way R&D spillovers, asymmetric information and government subsidies to explore their dynamic effects on price equilibrium.

The contents of this paper are arranged as follows. Relevant literatures in two fields is reviewed in Section: Literature review. A two-stage Stackelberg-Bertrand game model with one-way R&D spillovers, bounded rationality and effective information is proposed in Model Section. Then the Equilibrium points and local stability section conducts theoretical analysis of the stable existence conditions of Nash equilibrium in R&D investment. Various tools including three-dimensional (3D) stable region, two-dimensional (2D) and 3D bifurcation diagrams, one-dimensional (1D) and 2D maximum Lyapunov exponent (MLE), 3D average profits, strange attractors, attraction basins, are used to simulate the dynamic complexity in Numerical simulation section, where management significances are also put forward. Finally, we present the conclusion and corresponding policy recommendations in the last section.

## Literature review

The literature relevant to this study mainly focuses on two aspects: one is the mixed games of oligopoly; the other is the R&D spillovers in mixed games.

### Mixed games of oligopoly

Cournot model [23] based on homogeneous products and Bertrand model [24] based on heterogeneous products have been paid much attention by scholars since they were proposed, some researchers found that mixed game is also common in reality, that is, some firms use output as a decision-making variable, while others use price. Ever since Bylka and Komar [25] first proposed the Cournot-Bertrand model in 1975, the research on mixed game has been growing. Singh and Vives [26] examined price and quantity duality in a differentiated duopoly, revealing that firms prefer quantity contracts for substitute goods and price contracts for complements when limited to these two binding agreements. Tremblay and Tremblay [27] analyzed product differentiation in the Cournot-Bertrand duopoly game, finding that homogeneous markets eliminate Bertrand firms, leaving Cournot firms pricing at marginal cost but constrained by potential Bertrand competition. Asproudis and Filippiadis [28] studied the abatement technology choice in three cases of duopoly Cournot, Bertrand and Cournot-Bertrand games, research showed that Bertrand firms adopt dirtier abatement tech than Cournot rivals, and quantity competition incentivizes greener choices due to output aggressiveness. The Stackelberg game, a dynamic sequential competition model in game theory, delineates strategic interactions between a “leader" (first-mover decision-maker) and a “follower" (second-mover responder) in market contexts. Given its capacity to characterize hierarchical leader-follower relationships prevalent across multiple real-world domains, this framework has been extensively applied in economic, managerial, and political analyses, with substantial scholarly attention directed toward Stackelberg competition in both duopolistic and oligopolistic market structures [29,30]. Early studies paid little attention to two-stage or multi-stage game, but the diversity of decision variables and multi-stage product generation in real economy force scholars and business managers to consider two-stage or multi-stage oligopoly game. Since D’Aspremont and Jacquemin’s AJ model [31] analyzed R&D and production cooperation scenarios in two-stage games, subsequent research on such frameworks has proliferated. Mathematical properties of Stackelberg-Cournot and Cournot-Stackelberg games have been studied in works [32,33], three or four stage oligopoly games [3,34] is also used in international trade and other fields. However, the aforementioned literatures are all based on complete rationality, ignoring bounded rationality. Although the information asymmetry in oligopoly games has attracted a lot of attention, it is still mostly analyzed under the background of completely rational or one-stage game.

### R&D spillovers in monopoly games

Since D’Aspremont and Jacquemin first studied the influence of R&D spillover on the Nash equilibrium of duopoly’s R&D and production stages, R&D spillover has been the focus up to now. Tesoriere [35] studied the influence of one-way R&D spillovers on game equilibrium under R&D symmetry, and found that only simultaneous R&D proves sustainable as a subgame-perfect Nash equilibrium, preventing endogenous asymmetry. Zhao and Ding [36] examined how spillovers affect innovation investments across leader-follower timing strategies in an enterprise cluster, revealed evolving cluster innovation patterns and proposed policy measures to optimize R&D dynamics. Zhang *et al*. [37] explored government-set minimum quality standards for duopolistic firms’ innovation under Cournot and Bertrand competition, analysis showed that Bertrand competition prompted stricter MQS than Cournot, boosting R&D investment. With the increasing attention paid to bounded rationality, more and more literatures embed R&D spillovers into boundedly rational games. Bischi and Lamantia discussed R&D externalities in a two-stage game [38,39] and pointed out that R&D spillovers was beneficial to stable equilibrium outputs [40]. Tu and Wang [41] developed a triopoly R&D game with bounded rationality and spillover effects, showing that excessive R&D adjustment triggered chaos. Zhou *et al*. [42] modeled a differentiated Cournot duopoly with R&D spillovers, revealing that flip and Neimark-Sacker bifurcations led to chaos. The spillover effects discussed in the aforementioned literature primarily focus on bidirectional spillovers between enterprises or unidirectional spillovers along vertical supply chains, while paying insufficient attention to unilateral spillovers among horizontal enterprises. In reality, technologically advanced enterprises that have accumulated substantial prior knowledge are often capable of identifying technological opportunities within clusters at an earlier stage, thereby initiating innovation leadership [36]. Moreover, unidirectional R&D spillovers between enterprises are closely associated with multiple factors including technological gaps, market dominance, and technology life cycles [9,36]. Consequently, this study differs from previous research in two primary aspects: First, this paper investigates unidirectional spillovers among horizontal enterprises under a bounded rationality framework. Although Long and Huang [9] examined one-way spillovers through a two-stage Stackelberg-Cournot game, their analysis was predicated on complete information in output competition, whereas this research employs an asymmetric information framework in price competition. Second, while existing studies predominantly address R&D spillovers among generic enterprises, they largely overlook the specific context of enterprise clusters. The manifestation of R&D spillover effects proves particularly significant among clustered enterprises due to their intensified interactions and knowledge proximity.

### Contribution statements

Compared to extant research, the contribution of our article is mainly reflected in three aspects. First, unlike conventional models that often examine R&D spillovers or government subsidies in isolation, this research innovatively integrates one-way R&D spillovers, effective information, and government subsidies into a unified dynamic two-stage Stackelberg-Bertrand framework, capturing interdependencies often overlooked in static or single-parameter analyses. Although the articles [4,17] explored asymmetric information with bounded rationality, what we differed from them was the analysis of two-stage game instead of one-stage. Second, one-way R&D spillovers in a two-stage Stackelberg-Bertrand game with bounded rationality are studied in our paper, while most of previous works are based on bilateral R&D spillovers or complete rationality. Although the literature [9] analyzed one-way R&D spillovers under bounded rationality, it analyzed a Stackelberg-Cournot game instead of the Stackelberg-Bertrand game. Third, our findings challenge conventional wisdom in two critical aspects. On one hand, we reveal that excessive product homogeneity under certain conditions predominantly induces price volatility and market disorder, whereas moderate R&D spillover effects, while marginally reducing the leader’s profitability, generate positive externalities at the cluster level-phenomena insufficiently explored in prior scholarship. On the other hand, the stabilizing mechanisms embodied in government subsidies and the leader’s strategic information dissemination contrast markedly with existing literature that predominantly conceptualizes subsidies as profit-enhancing instruments rather than stabilization tools.

## Stackelberg-Bertrand game model with one-way R&D spillovers and asymmetric information

This study examines a duopolistic market structure within an industrial cluster, where two strategically distinct firms produce horizontally differentiated goods. Firm 1 operates as the R&D leader specializing in product 1, while firm 2 functions as the R&D follower manufacturing product 2. The strategic interaction unfolds through a two-stage Stackelberg-Bertrand hybrid game framework. During the initial phase, a sequential non-cooperative R&D competition is established under Stackelberg leadership dynamics. The leader first determines its optimal R&D expenditure for autonomous innovation. Subsequently, the follower, upon observing the leader’s innovation investment, strategically allocates its R&D resources towards derivative innovation activities. Notably, this phase incorporates governmental innovation incentives through proportional R&D subsidy allocations designed to stimulate technological advancement. The subsequent phase transitions to a Bertrand-Nash equilibrium model, where both firms engage in strategic price competition within the differentiated product market. This dual-stage game-theoretic framework enables comprehensive analysis of sequential decision-making processes encompassing both innovation investment strategies and market competition mechanisms.

We posit that the market demand function is characterized by:


$$q_{i}=a-p_{i}+dp_{j},\;i,j=1,2,\;i\neq j,$$

where *p*_*i*_ and *q*_*i*_ denote the price and output of product *i*, respectively. Here, *a* > 0 represents the potential market demand, and d∈[0,1] signifies the degree of substitution between products 1 and 2, with a higher value of *d* indicating greater fungibility or homogeneity between them. Furthermore, we assume that the unit production cost for both firms before any R&D engagement is denoted by *c*, constrained such that 0 < *c* < *a*.

In the Stackelberg R&D phase, since firm 2 learns imitative innovation from rival firm 1, R&D spillovers can only flow from firm 1 to firm 2 in one direction, then firm 1 cannot obtain R&D spillovers from firm 2. Consequently, after R&D activities, the unit cost of product 1 is $c_{1}=c-\beta_{1}\sqrt{x_{1}}$, while the unit cost of product 2 is $c_{2}=c-\beta_{2}(\sqrt{x_{2}}+\theta \sqrt{x_{1}})$. More specifically, $\beta_{i}\sqrt{x_{i}}(i=1,2)$ denotes the production function of firm *i*’s R&D input, which is the inverse mapping of R&D cost function in AJ model [31], and satisfies the law of diminishing marginal returns; *x*_*i*_ is firm *i*’s R&D input; $\beta_{i}$ represents firm *i*’s R&D efficiency, larger $\beta_{i}$ means higher R&D efficiency; θ measures the degree of R&D spillovers, θ=0 shows that R&D achievements are strictly protected and the technological innovation is difficult to spill freely between firms, while θ=1 indicates that R&D achievements are obtained for free by the follower due to full spillovers. Considering the R&D subsidies given by the government to innovative firms, we introduce the subsidy ratio *s*, then firm *i*’s R&D input cost function after receiving subsidies is (1−*s*)*x*_*i*_.

We use backward induction to determine R&D investment. In the second stage of Bertrand game, two firms determine their product prices according to profit maximization. Based on above assumptions, the profits of firm 1 and firm 2 are as follows:

$$\pi_{1}=(p_{1}-c+\beta_{1}\sqrt{x_{1}})(a-p_{1}+dp_{2})-(1-s)x_{1}\;\pi_{2}=(p_{2}-c+\beta_{2}\sqrt{x_{2}}+\beta_{2}\theta \sqrt{x_{1}})(a-p_{2}+dp_{1})-(1-s)x_{2}$$ (1)

The marginal profit on price can be obtained from Eq (1) as:

$$\frac{\partial \pi_{1}}{\partial p_{1}}=a-2p_{1}+dp_{2}+c-\beta_{1}\sqrt{x_{1}}\;\frac{\partial \pi_{2}}{\partial p_{2}}=a-2p_{2}+dp_{1}+c-\beta_{2}(\sqrt{x_{2}}+\theta \sqrt{x_{1}})$$ (2)

Then we can see that the equilibrium prices is:

$$p_{1}^{B}=\frac{\left(2+d)(a+c)-2\beta_{1}\sqrt{x_{1}}-d\beta_{2}(\sqrt{x_{2}}+\theta \sqrt{x_{1}}\right)}{4-d^{2}}\;p_{2}^{B}=\frac{\left(2+d)(a+c)-d\beta_{1}\sqrt{x_{1}}-2\beta_{2}(\sqrt{x_{2}}+\theta \sqrt{x_{1}}\right)}{4-d^{2}}$$ (3)

To ensure that the equilibrium price has practical significance, the relevant parameters need to meet Eq (4):

$$\left\{ \begin{array}{c} (2+d)(a+c)-2\beta_{1}\sqrt{x_{1}}-d\beta_{2}(\sqrt{x_{2}}+\theta \sqrt{x_{1}})>0\\ {}[11pt](2+d)(a+c)-d\beta_{1}\sqrt{x_{1}}-2\beta_{2}(\sqrt{x_{2}}+\theta \sqrt{x_{1}})>0 \end{array}\right.$$ (4)

In the first Stackelberg R&D stage, we would calculate the R&D input by substituting the equilibrium prices Eq (3) into the profit function (Eq 1). Since firms invest in R&D activities successively, we let $\frac{\partial \pi_{2}}{\partial x_{2}}=0$ first and calculate out the best reply function of firm 2, which is showed in the following form:

$$x_{2}={\left\{\frac{\beta_{2}(2-d^{2})[(2-d^{2})\beta_{2}\theta \sqrt{x_{1}}+(2+d)(a-c+cd)-d\beta_{1}\sqrt{x_{1}}]}{(1-s)(d^{2}-4{)}^{2}-\beta_{2}^{2}(2-d^{2}{)}^{2}}\right\}}^{2}$$ (5)

Then substitute Eq (5) into the first equation of Eq (1), and let $\frac{\partial \pi_{1}}{\partial x_{1}}=0$, firm 1’s R&D input $x_{1}^{*}$ can be got; next step by step, substitute $x_{1}^{*}$ into Eq (5) to get $x_{2}^{*}$, substitute $x_{1}^{*},x_{2}^{*}$ into Eq (3) to obtain the final equilibrium prices ($p_{1}^{*},p_{2}^{*}$). The specific values can be seen in Appendix.

The above analysis is based on the assumption that the enterprises are completely rational. However, in the actual economic society, affected by the limited information and the uncertainty of the society itself, the enterprises are boundedly rational, and the decision-making is often a process of constant adjustment, rather than one step. Based on this, it is assumed that firm 2 adopts GD mechanism to adjust the price, he will increase (reduce) the price when the marginal profit is positive (negative), the dynamic adjustment process can be described as follows:

$$p_{2}(t+1)=p_{2}(t)+vp_{2}(t)\left[a-2p_{2}(t)+dp_{1}(t)+c-\beta_{2}(\sqrt{x_{2}}+\theta \sqrt{x_{1}})\right],$$ (6)

where v∈[0,1] denotes the speed of price adjustment.

We assume firm 1 uses a more rational estimation mechanism. First, firm 1 will collect relevant market information before setting the price, so as to obtain as much information as possible about the current product price of firm 2, and makes its estimation based on this information, thereby we name firm 1 as a rational estimation player. Referring to the literature [17], we introduce an effective information parameter λ(λ∈[0,1]) to represent the degree of information acquisition, λ=0 means that firm 1 doesn’t know the current price information of firm 2 at all, while λ=1 indicates that the current price is known to firm 1. Therefore firm 1’s estimation of firm 2’s price at period t+1 is $p_{2}^{e}(t\;+\;1)=(1\;-\;\lambda )p_{2}(t)\;+\;\lambda p_{2}(t\;+\;1)$. We assume that firm 1 adopts the rational estimation method to determine the price, then his price evolution dynamics can be expressed as follows:

$$p_{1}(t+1)=\frac{a+dp_{2}^{e}(t+1)+c-\beta_{1}\sqrt{x_{1}}}{2}.$$ (7)

Combining Eqs (6) and (7), a price discrete system with asymmetric information and bounded rationality can be described as:

$$\left\{ \begin{array}{c} p_{1}(t+1)=\frac{a+d\left[(1-\lambda )p_{2}(t)+\lambda p_{2}(t+1)\right]+c-\beta_{1}\sqrt{x_{1}}}{2}\\ \left[13pt]p_{2}(t+1)=p_{2}(t)+vp_{2}(t)[a-2p_{2}(t)+dp_{1}(t)+c-\beta_{2}(\sqrt{x_{2}}+\theta \sqrt{x_{1}})\right] \end{array}\right.$$ (8)

## Equilibrium points and local stability

Let $p_{i}(t+1)=p_{i}(t)$ in system (8), we can get two equilibrium points :


$$E_{0}\left(\frac{a+c-\beta_{1}{\sqrt{x_{1}}}^{*}}{2},0\right),E_{1}(p_{1}^{*},p_{2}^{*}).$$

Obviously *E*_0_ is a boundary equilibrium point, *E*_1_ is the Nash equilibrium desired by the duopoly in practice. In order to analyze the stability of equilibrium points, we need to obtain the Jacobian matrix of the two-dimensional system (8) first, which is found as follows:


$$J=\left[\begin{array}{cc} \frac{v\lambda d^{2}p_{2}}{2} & \frac{d(1-\lambda +\lambda J_{22})}{2}\\ {}[10pt]vdp_{2} & J_{22} \end{array}\right]$$

where $J_{22}=1+v\left[a+c-4p_{2}+dp_{1}-\beta_{2}(\sqrt{x_{2}}+\theta \sqrt{x_{1}})\right]$.

**Proposition 1**. ***The boundary equilibrium point *E****_***0***_ ***of system (***8***) is unstable*.**

Proof. At point *E*_0_, its Jacobian matrix is


$$J(E_{0})=\left[\begin{array}{cc} 0 & \frac{d(1-\lambda +\lambda J_{22}^{*})}{2}\\ {}[10pt]0 & J_{22}^{*} \end{array}\right]$$

where $J_{22}^{*}=1+\frac{v}{2}\left[(2+d)(a+c)-d\beta_{1}{\sqrt{x_{1}}}^{*}-2\beta_{2}({\sqrt{x_{2}}}^{*}+\theta {\sqrt{x_{1}}}^{*})\right]$.

It’s clear that the matrix $J(E_{0})$ has two eigenvalues: $\varphi_{1}=0$, $\varphi_{2}=J_{22}^{*}$, and it’s to know $\varphi_{2}>1$ under condition (4), then proposition 1 is proved.

In reality, the boundary equilibrium point means that an enterprise has withdrawn from the market, and the market has become a complete monopoly market composed of an oligopolist. This is not conducive to the healthy development of the market, and may harm consumer rights and social welfare. Therefore, Nash equilibrium point *E*_1_ is the stable equilibrium expected by the monopoly enterprises. Below we will explore the conditions for the stable existence of *E*_1_.

**Proposition 2**. ***On the premise that firm 1 uses a rational estimation expectation and firm 2 adopts a GD expectation, $E_{1}(p_{1}^{*},p_{2}^{*})$ would be locally asymptotically stable if $\lambda >\lambda^{*}=\frac{(4+d^{2})vp_{2}^{*}-4}{2vd^{2}p_{2}^{*}}$. System (***8***) may experiences chaos and period-doubling bifurcations when $\lambda <\lambda^{*}$, passes through a flip bifurcation at $\lambda =\lambda^{*}$, and eventually becomes stable when $\lambda >\lambda^{*}$.***

Proof. As for Nash equilibrium point *E*_1_, its Jacobian matrix is:


$$J=\left[\begin{array}{cc} \frac{v\lambda d^{2}p_{2}^{*}}{2} & \frac{d\left[1-\lambda +\lambda (1-2vp_{2}^{*})\right]}{2}\\ {}[10pt]vdp_{2}^{*} & 1-2vp_{2}^{*} \end{array}\right].$$

We suppose the characteristic equation of $J(E_{1})$ has the following form:


$$P(\varphi )=\varphi^{2}-Tr\varphi +Det=0,$$

where *Tr* is the trace and *Det* is the determinant of $J(E_{1})$; and accordingly,


$$Tr(J(E_{1}))=1-2vp_{2}^{*}+\frac{v\lambda d^{2}p_{2}^{*}}{2},\;Det(J(E_{1}))=\frac{v(\lambda -1)d^{2}p_{2}^{*}}{2}.$$

It is obvious that $Det(J(E_{1}))<0$ under condition λ∈[0,1], hence $Tr(J(E_{1}){)}^{2}-4Det(J(E_{1}))>0$, the characteristic equation has two real roots. The sufficient and necessary conditions that the Nash equilibrium point *E*_1_ is locally stable are given by Jury’s conditions:

$$\left\{ \begin{array}{c} (i):1+Tr\left(J(E_{1})\right)+Det\left(J(E_{1})\right)>0\\ {}[10pt](ii):1-Tr\left(J(E_{1})\right)+Det\left(J(E_{1})\right)>0\\ {}[10pt](iii):1-Det\left(J(E_{1})\right)>0 \end{array}\right.$$ (9)

Eq (9) gives the stable region in which the Nash equilibrium exists, failure to hold any of these conditions can lead to bifurcation and even chaos. We plug the exact values of $Tr(J(E_{1}))$ and $Det(J(E_{1}))$ into Eq (9), which can be written as Eq (10):

$$\left\{ \begin{array}{c} (i):2-2vp_{2}^{*}+\frac{(2\lambda -1)vd^{2}p_{2}^{*}}{2}>0\\ {}[10pt](ii):\frac{(4-d^{2})vp_{2}^{*}}{2}>0\\ {}[10pt](iii):1-\frac{(\lambda -1)vd^{2}p_{2}^{*}}{2}>0 \end{array}\right.$$ (10)

With λ∈[0,1] and d∈[0,1], inequalities (*ii*)(*iii*) in Eq (10) obviously always hold, so the Jury’s conditions is equivalent to only having to satisfy inequality (*i*), namely, Eq (11):

$$2-2vp_{2}^{*}+\frac{(2\lambda -1)vd^{2}p_{2}^{*}}{2}>0$$ (11)

After ascertaining the stable region of Nash equilibrium point *E*_1_ given by the inequality in Eq (11), we then study the influence of certain parameters, such as price adjustment speed and effective information, on the local stability of the equilibrium point.

The critical value of effective information $\lambda^{*}=\frac{(4+d^{2})vp_{2}^{*}-4}{2vd^{2}p_{2}^{*}}$ can be easily calculated from Eq (11), and thus Proposition 2 can be verified by combining with the stability theory of discrete systems.

According to the same calculation process, the influence of price adjustment speed on the local stability of the equilibrium point is also easy to analyze, see Proposition 3.

**Proposition 3**. ***On the premise that firm 1 uses a rational estimation expectation and firm 2 adopts a GD expectation, $E_{1}(p_{1}^{*},p_{2}^{*})$ would be locally asymptotically stable if $v<v^{*}=\frac{4}{\left[4-(2\lambda -1)d^{2}\right]p_{2}^{*}}$. The larger*** *v* ***is, the more likely system (8) is to be unstable. The system will go from stable to two-period bifurcation at $v=v^{*}$, undergo period-doubling bifurcations, and finally fall into chaos as*** *v* ***increases.***

In this section, we analyze the conditions for the stability of Nash equilibrium, give the stability region, and discuss the influence of parameters v,λ on the stability of discrete system. However, due to the complexity of the equilibrium price $p_{2}^{*}$ (data in S1 File), it is impossible to theoretically study the influence of parameters $d,s,\theta ,\beta_{1},\beta_{2}$ on the local stability of Nash equilibrium, so next section we seek to explore this further through numerical simulation.

## Numerical simulation and management significance

In this section, numerical simulations conducted via MATLAB will demonstrate the dynamic complexity of the system (8), the evolution trajectory of the influence of parameters with different values on the stability of equilibrium points will also be described. Various tools, such as 1D, 2D and 3D bifurcation diagrams, 1D and 2D maximum Lyapunov exponent (MLE), 3D average profits, strange attractors, attraction basins, sensitivity to initial conditions, are employed to exhibit the existence of stability, bifurcation and chaos. We also accordingly put forward the management significance in practice. To ensure sufficient potential market demand capacity, we set *a* = 4, which is significantly larger than other parameters. For instance, the unit costs for both firms are set as *c* = 1. Furthermore, to demonstrate firm 1’s R&D leadership position, we configure its R&D efficiency as $\beta_{1}=0.5$, while the follower firm 2’s R&D efficiency is set at $\beta_{2}=0.2$. Considering government incentives for corporate R&D, we assume a 20% subsidy rate on enterprises’ R&D investments, namely, *s* = 0.2. Additionally, we set the degree of effective information mastery by firm 1 over firm 2 to λ=0.2, and establish the unidirectional R&D spillover effect from firm 1 to firm 2 as θ=0.2.

**Fig 1:**
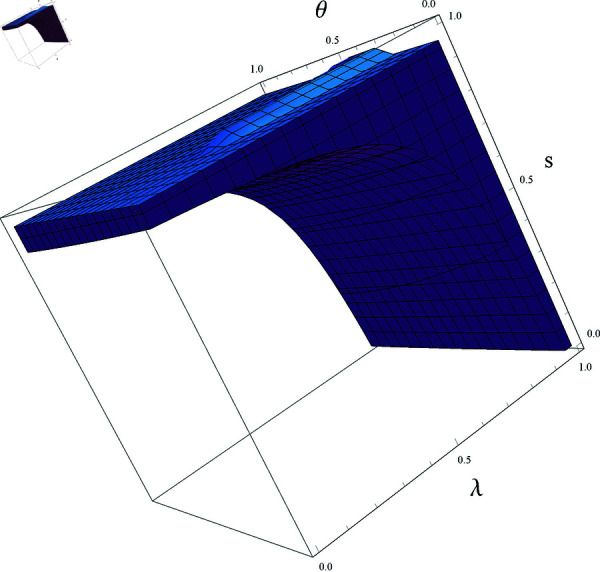
3D stable region of system (8).

Fig 1 illustrates the stable region enclosed by λ,θ,s, with other parameters set as $\left(a,c,d,v,\beta_{1},\beta_{2})=(4,1,0.95,0.28,0.5,0.2\right)$. As can be observed from the figure, with the other two parameters held constant, the value of θ always remains within the stable region. Moreover, as *s* or λ gradually increases, the stable region also expands. This implies that a higher government R&D subsidy, or more effective information about the follower’s product prices in the hands of the R&D leader, is more conducive to price stability.

**Fig 2:**
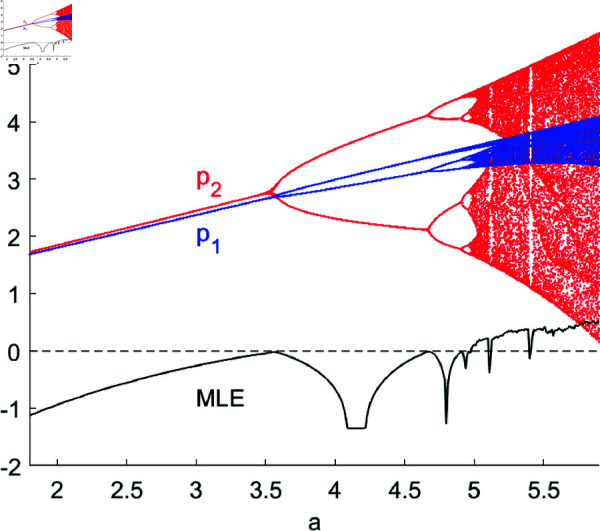
The bifurcation diagram and MLE of system (8) with respect to a. Other parameters are set as: $\left(c,d,s,\beta_{1},\beta_{2},\theta ,v)=(1,0.4,0.2,0.5,0.2,0.2,0.35\right)$.

**Fig 3:**
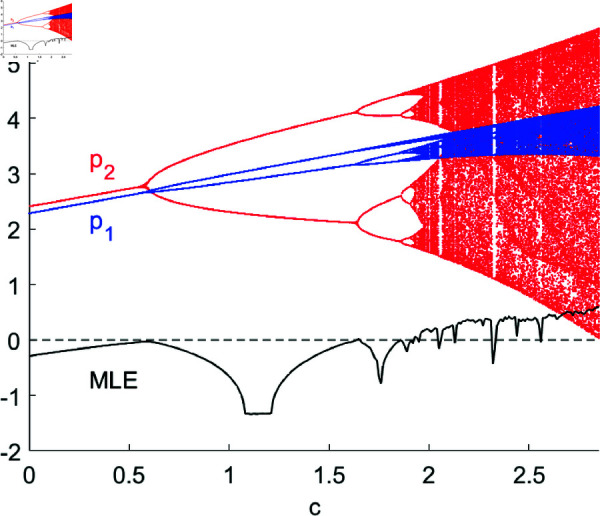
The bifurcation diagram and MLE of system (8) with respect to c. Other parameters are set as: $\left(a,d,s,\beta_{1},\beta_{2},\theta ,v)=(4,0.4,0.2,0.5,0.2,0.2,0.35\right)$.

Figs 2 and 3 depict the bifurcation diagrams of system (8) with respect to parameters *a* and *c*, respectively. As *a* and *c* gradually increase, the product price initially rises when *a* < 3.5667 and *c* < 0.5924, subsequently entering into higher-order bifurcations such as period-doubling, period-quadrupling, period-octuplication, etc., leading to price fluctuations. Eventually, the system enters a chaotic state. MLE is also calculated and plotted in the diagrams, where a positive (negative) MLE indicates that the system is in a chaotic state (stable or bifurcated state), and MLE=0 indicates that period-doubling bifurcations are occurring. In reality, as the potential market capacity increases, product prices rise due to supply shortages. However, when the potential market capacity becomes sufficiently large, collective optimism or panic can easily form. The herding behavior driven by rising prices and the resulting bubbles may lead to a rapid decline in prices in the subsequent stage, causing significant price swings and even turmoil. When the unit cost of a product increases moderately, enterprises will appropriately raise prices to maintain profitability. Yet, when the unit cost becomes excessively high, passing on the high cost to consumers will reduce demand and lead to lower prices. Conversely, excessively low prices will stimulate consumption and drive up product prices, resulting in price swings.

**Fig 4:**
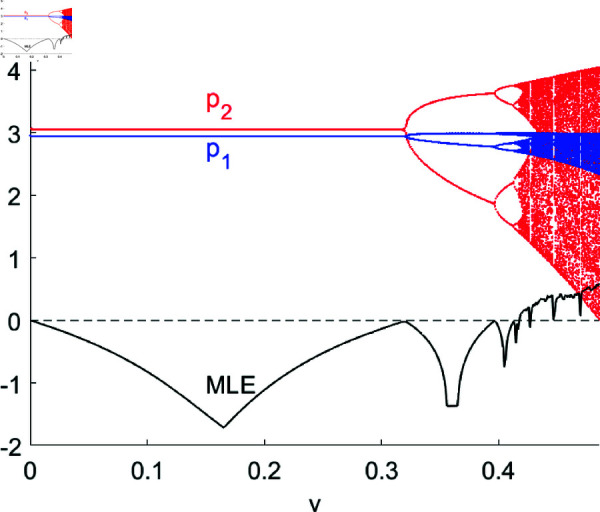
The bifurcation diagram and MLE of system (8) with respect to v.

Fig 4 presents the bifurcation diagram and MLE of system (8) concerning *v* with others parameters fixed as $\left(a,c,d,s,\beta_{1},\beta_{2},\lambda ,\theta )=(4,1,0.4,0.2,0.5,0.2,0.2,0.2\right)$. As can be seen from Fig 4, system (8) stays stable at Nash Equilibrium point *E*_1_(2.9423,3.0496) when $v<v^{*}=0.3202$, crosses the flip bifurcation surface at $v=v^{*}$, bifurcates from Nash Equilibrium point when *v* > ${\text{v}}^{*}$, experiences period doubling bifurcations and finally fall into chaos. Firms should maintain a moderate pace of price adjustments, as substantial adjustments may lead to market turmoil.

**Fig 5:**
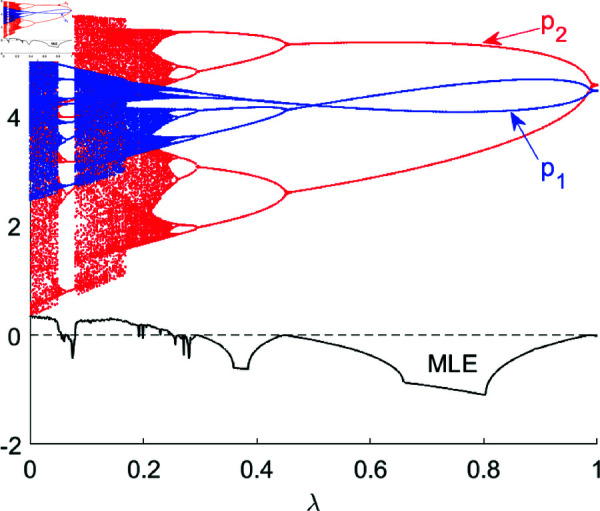
The bifurcation diagram and MLE of system (8) with respect to λ.

Fig 5 is the bifurcation diagram of system (8) with respect to λ, with other parameters set as $\left(a,c,d,s,v,\beta_{1},\beta_{2},\theta )=(4,1,0.95,0.2,0.28,0.5,0.2,0.2\right)$. When the effective information λ is small, the system is in a chaotic state. As λ gradually increases, the enterprises’ prices escape from the disordered state and enters into a doubly periodic bifurcation state. The system finally converges to the Nash Equilibrium point *E*_1_(4.4731,4.5761) when $\lambda >\lambda^{*}=0.9865$. This indicates that the more information the R&D leader obtains about the follower’s current product price, the more stable the prices become.

**Fig 6:**
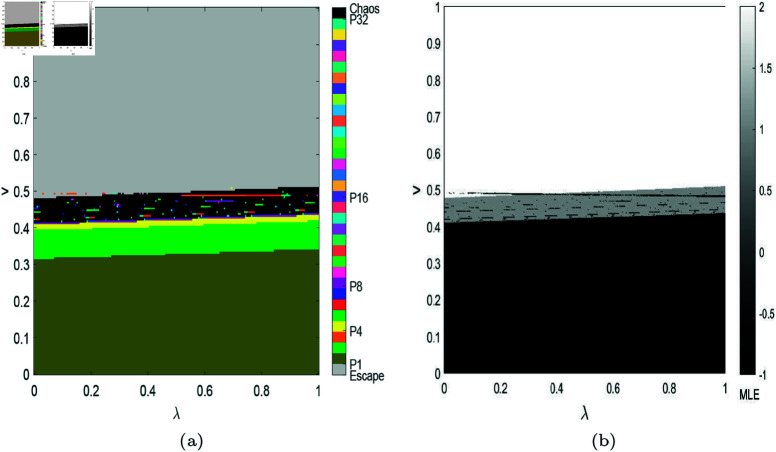
(a) shows 2D bifurcation diagram in the (λ,v) plane in system (8), other parameters are set as: $\left(a,c,d,s,\beta_{1},\beta_{2},\theta )=(4,1,0.4,0.2,0.5,0.2,0.2\right)$. (b) shows the corresponding 2D MLE.

Compared with 1D bifurcation diagram, 2D bifurcation diagram is more advantageous in numerical simulation of the complexity of nonlinear systems, and we can assign different colors to the regions where different periodic bifurcations are. In Fig 6(a), we depict 2D bifurcation diagram in the (λ,v) plane, where brown areas indicate system (8) stays stable, green, orange, yellow, dark green, red, blue and purple regions indicate that the system is in a 2-8 period bifurcation respectively, black means a chaotic state and gray indicates the escape area. As can be seen from Fig 6(a), when *v* is small, the value of effective information does not affect the system’s stability, and the Nash equilibrium price always exists; when *v* is relatively large, the system will be in chaos. We also calculated the corresponding MLE in Fig 6(b), MLE < 0 signifies that system (8) is in a stable or period-doubling bifurcation state, while MLE ∈(0,2) corresponds to the chaotic region and MLE >2 corresponds to the escape region.

**Fig 7:**
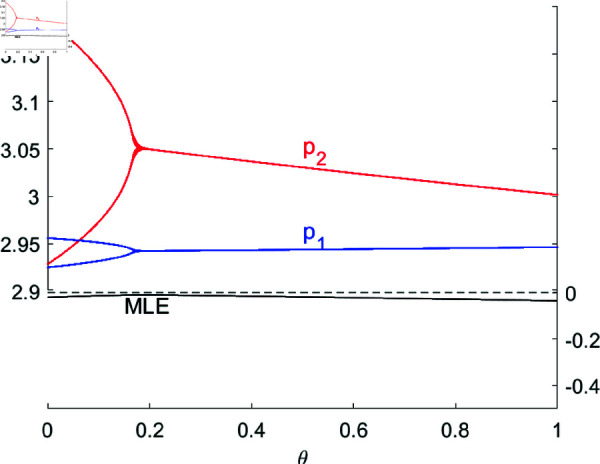
The bifurcation diagram with respect to θ.

**Fig 8:**
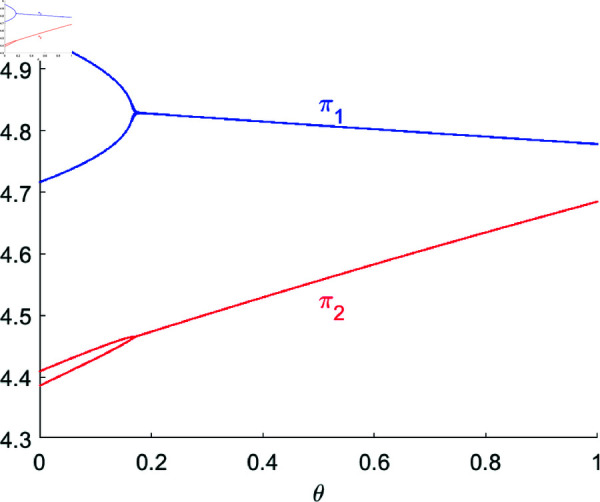
Profits of firms with respect to θ.

Fig 7 shows the bifurcation diagram of system (8) concerning R&D spillovers θ with $\left(a,c,d,s,v,\beta_{1},\beta_{2},\lambda )=(4,1,0.4,0.2,0.32,0.5,0.2,0.2\right)$. When θ is small ($\theta <\theta^{*}=0.1668$), system (8) is in a 2-period bifurcation state; as θ increase, the system tends to fixed point *E*_1_(2.9422,3.0518), and the price of product 2 is gradually decreasing, while the price of product 1 is almost unchanged. At the meantime, Fig 8 shows that the profit of R&D leader (firm 1) is also declining, while the profit of R&D follower (firm 2) is rapidly increasing due to the free acquisition of R&D spillovers. Therefore, in a cluster innovation environment where the leader independently innovates and the follower imitates innovation, an increase in one-way R&D spillovers benefits the profit increase of the follower, contributes to the overall revenue enhancement of the cluster, but harms the interests of the leader, thereby somewhat suppressing their enthusiasm.

**Fig 9:**
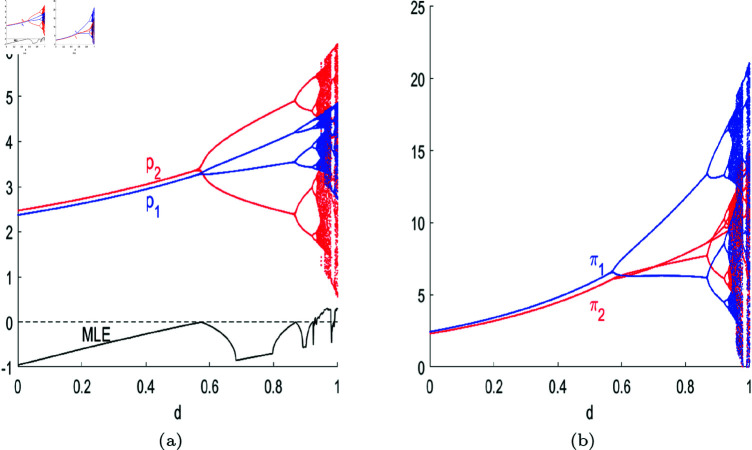
(a) The bifurcation diagram of system (8) with respect to *d.* Other parameters are $\left(a,c,s,v,\beta_{1},\beta_{2},\lambda ,\theta )=(4,1,0.2,0.28,0.5,0.2,0.2,0.2\right)$. (b) The corresponding profits of firms.

**Fig 10:**
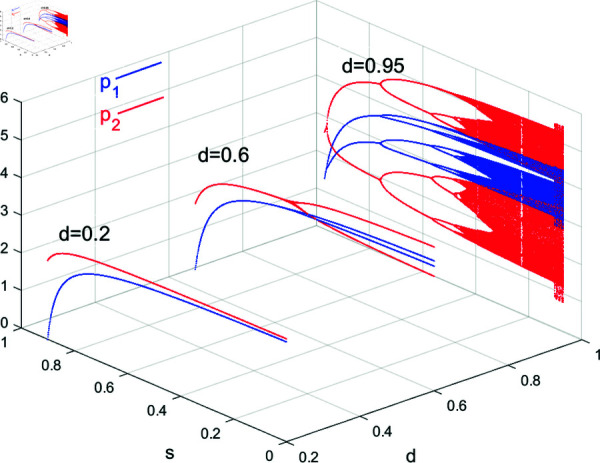
3D bifurcation diagram of system (8) with respect to *s* and *d*.

Fig 9 shows the bifurcation diagram of system (8) with respect to *d*. As can be seen from Fig 9(a), Nash equilibrium point *E*_1_ is locally stable for *d*<d^*^ = 0.5741; with *d* increasing, system (8) goes through 2-period bifurcation at ${\text{d}}^{*}$, 4-period bifurcation at *d* = 0.8684, and higher-order bifurcations until it ends in chaos. It can be seen that the higher the substitutability of products, that is, the more homogeneous the products, the more likely the market is to fall into disorder, moderate product differentiation is conducive to the improvement of individual enterprise’s profits and the overall cluster, which can be illustrated in Fig 9(b). Fig 10 simulates the 3D bifurcation diagram of system (8) concerning *d* and *s*, while other parameters are $\left(a,c,v,\beta_{1},\beta_{2},\lambda ,\theta )=(4,1,0.28,0.5,0.2,0.2,0.2\right)$. System (8) starts with chaotic or bifurcated state, it will converge to Nash Equilibrium when *s*>s^*^ = 0.8909 with *d* = 0.95 and *s*>s^*^ = 0.5089 with *d* = 0.6. When the value of *d* is small, namely *d* = 0.2, Nash Equilibrium price is always locally stable. It can be seen that the higher the government subsidies, the more stable the market become. When the post-R&D products are highly homogeneous, substantial government subsidies contribute to the stability of product prices; when there is a certain degree of differentiation in the post-R&D products, prices tend towards stability, and the higher the government subsidies, the lower the product prices.

**Fig 11:**
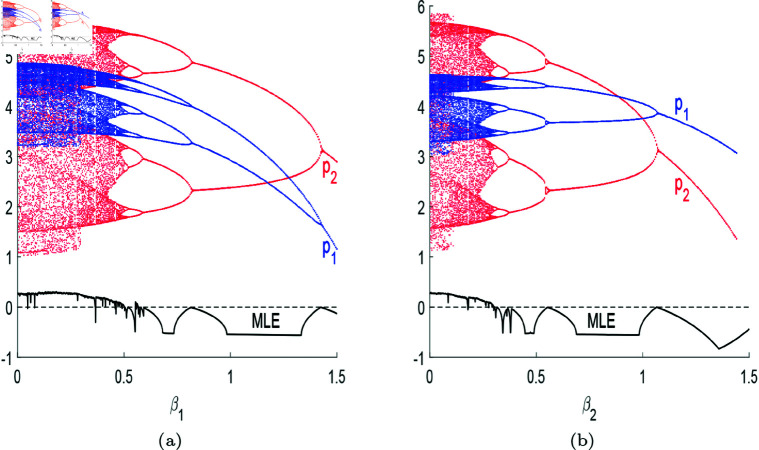
(a) The bifurcation diagram of system (8) with respect to $\beta_{1}$. Other parameters are $\left(a,c,d,s,v,\beta_{2},\lambda ,\theta )=(4,1,0.95,0.2,0.28,0.3,0.2,0.2\right)$. (b) The bifurcation diagram of system (8) with respect to $\beta_{2}$. Other parameters are $\left(a,c,d,s,v,\beta_{1},\lambda ,\theta )=(4,1,0.95,0.2,0.28,0.5,0.2,0.2\right)$.

Fig 11 shows the bifurcation diagram of the pricing system with respect to $\beta_{1},\beta_{2}$. As can be seen, with the increase in $\beta_{1},\beta_{2}$, the system will experience chaos, bifurcation, and eventually move towards stability. When the R&D efficiency is high, there exists a Nash equilibrium in the prices of post-R&D products, which will gradually decrease. An increase in the R&D efficiency of the leader will significantly reduce the price of its post-R&D products, making them more competitive in the market. Similarly, as the R&D efficiency of the follower gradually increases, the reduction in its product prices will also be greater than that of the leader’s price reduction.

**Fig 12:**
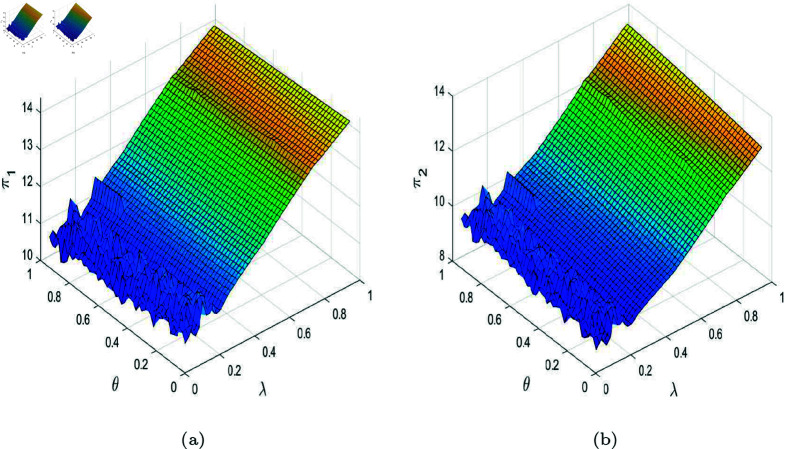
(a) Average profit of firm 1 with other parameters set as $\left(a,c,d,s,v,\beta_{1},\beta_{2})=(4,1,0.95,0.2,0.28,0.5,0.2\right)$. (b) Average profit of firm 2.

**Fig 13:**
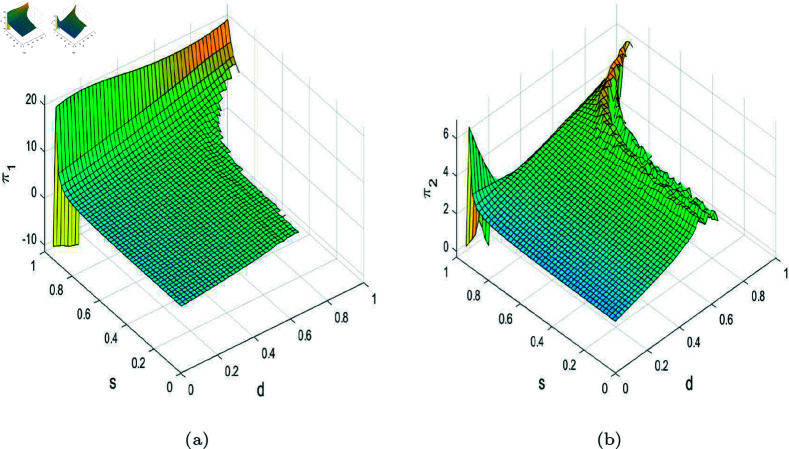
(a) Average profit of firm 1 with other parameters set as $\left(a,c,v,\beta_{1},\beta_{2},\lambda ,\theta )=(4,1,0.4,0.5,0.2,0.2,0.2\right)$. (b) Average profit of firm 2.

**Fig 14:**
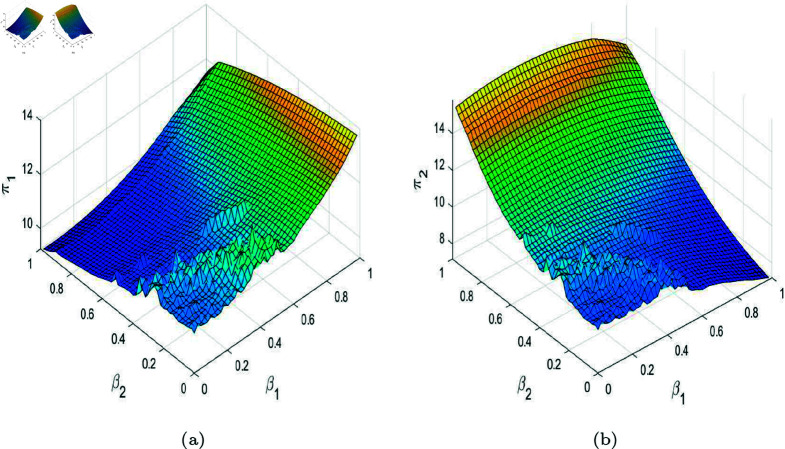
(a) Average profit of firm 1 with other parameters set as (a,c,d,s,v,λ,θ)=(4,1,0.95,0.2,0.28,0.2,0.2). (b) Average profit of firm 2.

In the stable state, the profits of the duopoly are equal to the Nash equilibrium profits. When the system becomes unstable, the profits of the enterprises cannot stabilize at the Nash equilibrium profits. In order to study the profits in unstable state, especially when entering chaos, we take the average of the profits of each enterprise over 200 periods as the average profit to measure the impact of system instability on their profits. Figs 12, 13, and 14 show 3D graphs of the average profits of enterprises under different parameters. In Fig 12, regardless of the value of R&D spillovers, as λ increases, the average profits of both firms tend to stabilize and show an increasing trend from a chaotic state, as described in Fig 5. When λ is small, their profits remain in a chaotic state, but when λ exceeds a certain threshold, the average profits of both firms increase with the rise in R&D spillovers. Fig 11 indicates that an increase in effective information and R&D spillovers contributes positively to enhancing the average profits of the enterprises. In Fig 13, when government subsidies stay within a certain range, the average profits of both firm increase slightly with the enhancement of product homogeneity. However, when the similarity of products is very high, prices can fall into a chaotic state, leading to fluctuations in average profits. When there is some differentiation in products, the profits of both firms increase slightly with an increase in government R&D subsidies. But when the proportion of government subsidies is very high, the average profits would experience significant volatility due to the prices falling into chaos, just as depicted in Fig 9. In Fig 14, when $\beta_{1}$ and $\beta_{2}$ are relatively small, the average profits of both firms oscillate due to chaotic price fluctuations. Their average profits would increase sharply with an improvement in their own R&D efficiency but decrease with an enhancement in the rival’s R&D efficiency. The evolutionary trajectory of average profits can be corroborated by Fig 11.

**Fig 15:**
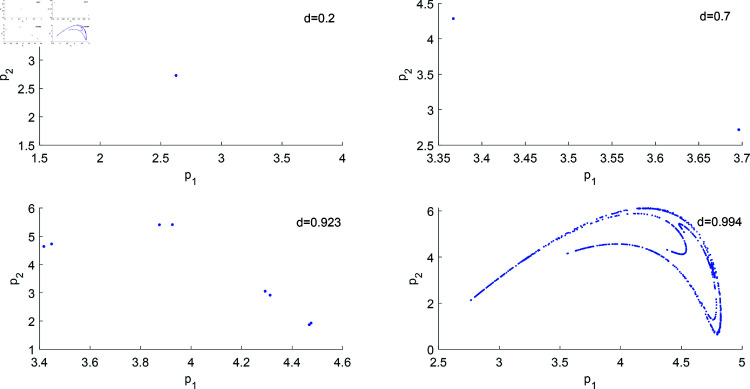
Attractors of system (8) for different values of d with $\left(a,c,s,v,\beta_{1},\beta_{2},\lambda ,\theta )=(4,1,0.2,0.28,0.5,0.2,0.2,0.2\right)$.

The final state of a nonlinear dynamic system can be represented by an attractor, which is the asymptotic behavior of a nonlinear system solution when the number of iterations approaches infinity. Fig 15 shows the attractors for different values of *d*. As can be seen, the increase in product substitutability leads to the complexity of the final behavior of system (8), and the evolution process presents a deterministic and irregular development, where “Determinism” is represented by bifurcation mode, and “Irregularity” is manifested as the complex structure inside the chaotic attractor. The evolution of attractors shows a high degree of complexity, both in terms of phenomenon development and internal mechanism, which means that the economic system is not accurate when forecasting.

**Fig 16:**
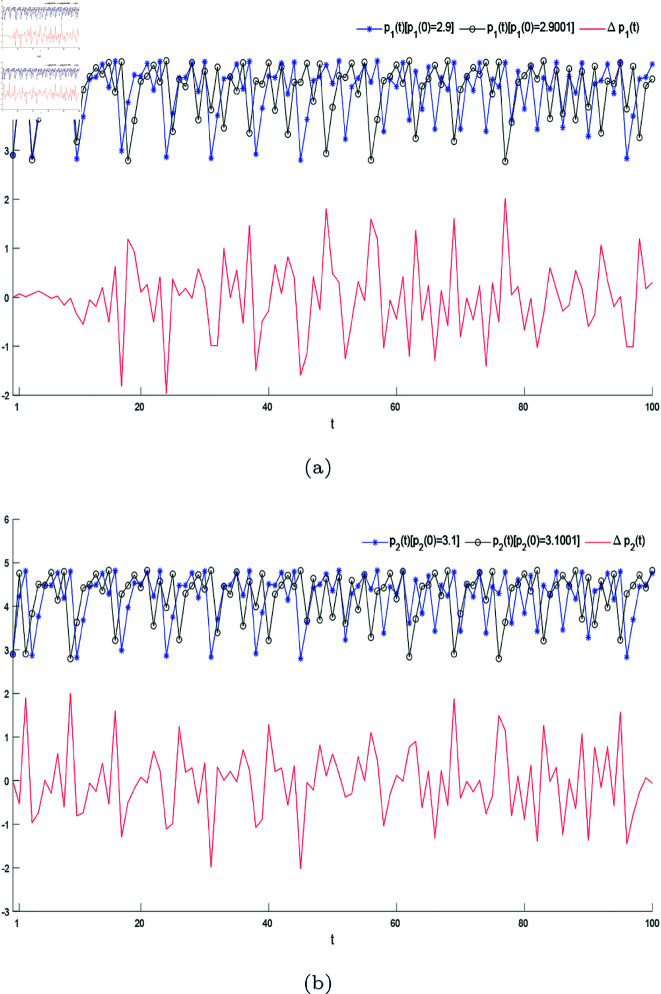
The sensitivity to initial values. (a) shows the different evolution trajectory of the initial price deviation of firm 1 from 0.0001 and the corresponding change of price gap with the increase of the number of iterations. (b) shows the different evolution trajectory of the initial price deviation of firm 2 from 0.0001 and the corresponding change of price gap with the increase of the number of iterations.

Initial value sensitivity is an important feature of chaos, which is manifested in that a small difference in initial value leads to a completely different final state of the system, it is also described vividly as the “butterfly effect”. Fig 16 demonstrates the sensitivity of system (8) to initial conditions. A small deviation 0.0001 from the initial prices $\left(p_{1}(0),p_{2}(0)=(2.9,3.1)\right)$ leads to a large difference after increasing number of iterations. The parameter settings in the Fig 16 are the same as in Fig 9(a).

**Fig 17:**
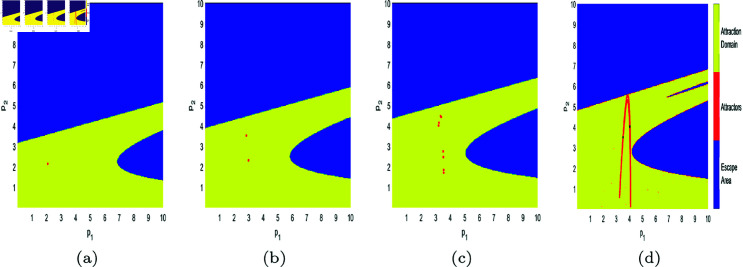
The attraction basin of system (8) with different values of *a.* (a) *a* = 2.5; (b) *a* = 4; (c) *a* = 4.95; (d) *a* = 5.9.

**Fig 18:**
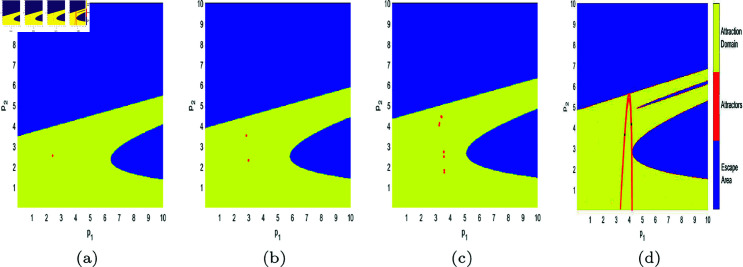
The attraction basin of system (8) with different values of c. (a) *c* = 0.2; (b) *c* = 1; (c) *c* = 1.885; (d) *c* = 2.85.

**Fig 19:**
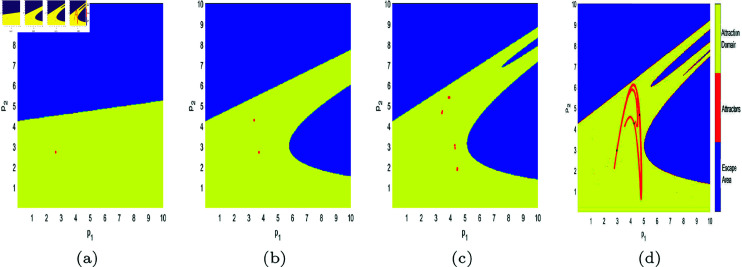
The attraction basin of system (8) with different values of d. (a) *d* = 0.2; (b) *d* = 0.7; (c) *d* = 0.923; (d) *d* = 0.994.

**Fig 20:**
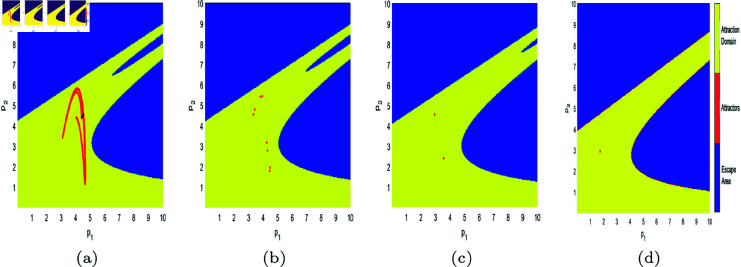
The attraction basin of system (8) with different values of s. (a) *s* = 0.2; (b) *s* = 0.42; (c) *s* = 0.8; (d) *s* = 0.89.

**Fig 21:**
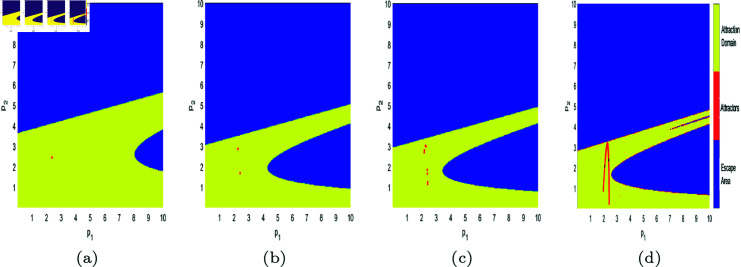
The attraction basin of system (8) with different values of v. (a) v=0.3; (b) v=0.46; (c) v=0.518; (d) v=0.59.

**Fig 22:**
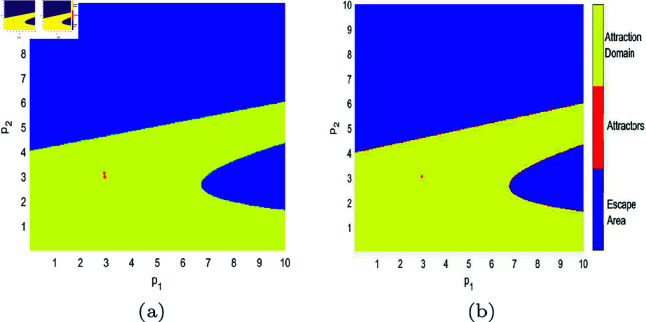
The attraction basin of system (8) with different values of θ. (a) θ=0.1; (b) θ=0.4.

**Fig 23:**
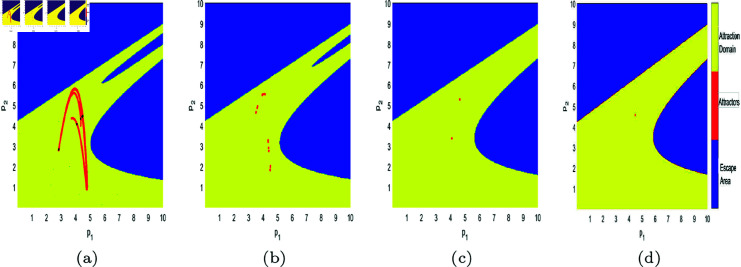
The attraction basin of system (8) with different values of λ. (a) λ=0.12; (b) λ=0.254; (c) λ=0.8; (d) λ=0.996.

In Figs 17, 18, 19, 20, 21, 22, and 23, where yellow, blue, and red set of points denote attraction domain, escape area, and attractors respectively, we introduce the basins of attraction to study the influence of parameters a,c,d,s,v,θ,λ on the attraction domain. Attraction domain is a set of initial prices that converge to the same attractor after a series of games. In an economic society, when all points in the attraction domain converge to an equilibrium point, then the equilibrium point is the Nash equilibrium point, and the price set of points contained in the attraction domain will tend to be stable after a series of iterations. If the initial price lies in the escape area, the system eventually falls into divergence. From these diagrams of attraction basins, the range of attraction domain gradually decreases with the increase of price adjustment speed *v* or product fungibility *d*, and the system moves from stability to bifurcation and chaos. In Figs 17(d), 18(d), 19(d) and 21(d), system (8) eventually converges to a chaotic state, where prices are unpredictable and markets fall into disarray. In contrast, the system tends from instability to stability as the parameter s,λ,θ increases in Figs 20, 22 and 23, where prices eventually converge to Nash Equilibrium. As can be seen from the above figures, in order to keep the market stable after multiple price games and reach Nash equilibrium, we should try our best to keep the values of *a*,*c*,*d*,*v* small and the values of s,λ,θ large. From the perspective of economics, in the price competition of innovative products composed of leading enterprises and following enterprises, in order to maintain the stability of the market, product differentiation should be kept as far as possible and the price adjustment speed should be small. Government subsidies for R&D activities, R&D spillovers, and effective information from leading firms contribute to the stability of product prices.

## Conclusion and policy implications

Combination of bounded rationality, asymmetric information and R&D spillovers were rarely discussed in the two-stage game in previous referrences. In this paper, we innovatively proposed a two-stage Stackelberg-Bertrand game model with boundedly rational duopoly. We analyzed the conditions for the existence of subgame perfect equilibrium and discussed the dynamic effects of R&D spillovers, effective information, R&D subsidies and product differentiation on the equilibrium price. Two important results are obtained through our analysis. First, the subgame perfect equilibrium is derived, along with the requisite condition for its stable existence and the corresponding stability region. The critical values of relevant parameters when the fold bifurcation occurs are also given. Second, we analyze the complexity of one-way R&D spillovers, government subsidy rate, effective information, product substitution and other parameters on the dynamic equilibrium; it is found that the higher the degree of product homogeneity, the more likely the discrete system will fall into bifurcation or chaos, and the price will appear oscillation and disorder; although one-way R&D spillovers is not conducive to R&D leaders, it is beneficial to the cluster as a whole; the higher the government subsidy rate or the more effective information available to R&D leaders, the more conducive to price stability.

In practice, to maintain the R&D stability and improve the profits of cluster enterprises, following measures can be adopted. Firstly, maintaining product differentiation is crucial. During price competition, the R&D followers will not be eliminated from the market due to weaker capabilities, and the profits of the R&D leaders will also increase due to differentiation. Secondly, a certain degree of R&D spillovers is beneficial for market stability when there are disparities in R&D capabilities among enterprises. Although profits of R&D leaders may be affected by R&D spillovers to some extent, this could lead to an overall increase in the cluster’s collective benefits. Thirdly, government subsidies in the R&D innovation process are conducive to regulating the prices of innovative products, especially in markets with severe product homogenization, where higher R&D subsidies can stimulate innovation among monopolistic enterprises when market prices fluctuate disorderly. Fourthly, the price adjustment speed of followers should not be too high. They should not make significant price adjustments based on higher marginal profits in the initial phase to avoid causing price shocks. Fifthly, the more effective information the R&D leaders master about the current prices of the R&D followers, the better they can make correct pricing decisions, thereby maintaining market stability.

This study exclusively examines unidirectional R&D spillovers within duopoly competition, omitting analysis of spillover dynamics among multiple oligopolistic entities and hybrid spillover effects across supply chain oligopolies. Furthermore, the spillover effects examined in this study remain confined to theoretical analysis, lacking empirical validation through practical applications such as carbon emission reduction technology development in green supply chains. Subsequent investigations will prioritize low-carbon R&D initiatives within green supply chain frameworks, aiming to facilitate the effective diffusion and application of carbon reduction.

## Supporting information

S1 FileAppendix: details exhibition for $x_{1}^{*},x_{2}^{*},p_{1}^{*},p_{2}^{*}$.(DOCX)
