# Supplementary material for: Dynamic complexity of Stackelberg-Bertrand game with one-way R&D spillovers, effective information and government subsidies
Source: PLoS One. 2025 Jul 28;20(7):e0328071. doi: 10.1371/journal.pone.0328071 (PMC12303324; doi:10.1371/journal.pone.0328071)
Supplement: S1 File — (DOCX) [file pone.0328071.s001.docx]

**Appendix: Details Exhibition for** $\boldsymbol{x}_{\boldsymbol{1}}^{\boldsymbol{*}}\boldsymbol{,}\boldsymbol{x}_{\boldsymbol{2}}^{\boldsymbol{*}}\boldsymbol{,}\boldsymbol{p}_{\boldsymbol{1}}^{\boldsymbol{*}}\boldsymbol{,}\boldsymbol{p}_{\boldsymbol{2}}^{\boldsymbol{*}}$

Since $x_{2}^{*}$ is related to $x_{1}^{*}$, and $p_{1}^{*},p_{2}^{*}$ are determined by $x_{1}^{*} and x_{2}^{*}$, we compute $x_{1}^{*}$ first, and its expression is as follows:

$$x_{1}^{*}=\frac{M_{1}^{2}}{M_{2}^{2}} ,$$

where

$M_{1}=\left( a-c+cd \right)\left( 4d-8s-4ds-2\beta_{2}^{2}d+2d^{2}s+d^{3}s-2\beta_{2}^{2}-2d^{2}-d^{3}+\beta_{2}^{2}d^{2}+\beta_{2}^{2}d^{3}+8 \right)\left( 8\beta_{1}s-8\beta_{1}+2\beta_{1}\beta_{2}^{2}+6\beta_{1}d^{2}-\beta_{1}d^{4}-3\beta_{1}\beta_{2}^{2}d^{2}+\beta_{1}\beta_{2}^{2}d^{4}+4\beta_{2}d\theta-6\beta_{1}d^{2}s+\beta_{1}d^{4}s-\beta_{2}d^{3}\theta-4\beta_{2}ds\theta+\beta_{2}d^{3}s\theta\right)$,

$M_{2}=\left( \beta_{2}^{2}d^{6}s^{2}-2\beta_{2}^{2}d^{6}s+\beta_{2}^{2}d^{6}-8\beta_{2}^{2}d^{4}s^{2}+16\beta_{2}^{2}d^{4}s-8\beta_{2}^{2}d^{4}+16\beta_{2}^{2}d^{2}s^{2}-32\beta_{2}^{2}d^{2}s+6\beta_{2}^{2}d^{2} \right)\theta^{2}+\left( 2\beta_{1}\beta_{2}^{3}d^{7}s-2\beta_{1}\beta_{2}^{3}d^{7}-14\beta_{1}\beta_{2}^{3}d^{5}s+14\beta_{1}\beta_{2}^{3}d^{5}+28\beta_{1}\beta_{2}^{3}d^{3}s-28\beta_{1}\beta_{2}^{3}d^{3}-16\beta_{1}\beta_{2}^{3}ds+16\beta_{1}\beta_{2}^{3}d+2\beta_{1}\beta_{2}d^{7}s^{2}-4\beta_{1}\beta_{2}d^{7}s+2\beta_{1}\beta_{2}d^{7}-20\beta_{1}\beta_{2}d^{5}s^{2}+40\beta_{1}\beta_{2}d^{5}s-20\beta_{1}\beta_{2}d^{5}+64\beta_{1}\beta_{2}d^{3}s^{2}-128\beta_{1}\beta_{2}d^{3}s+64\beta_{1}\beta_{2}d^{3}-64\beta_{1}\beta_{2}ds^{2}+128\beta_{1}\beta_{2}ds-64\beta_{1}\beta_{2}d \right)\theta+\beta_{1}^{2}\beta_{2}^{4}d^{8}-6\beta_{1}^{2}\beta_{2}^{4}d^{6}+13\beta_{1}^{2}\beta_{2}^{4}d^{4}-12\beta_{1}^{2}\beta_{2}^{4}d^{2}+4\beta_{1}^{2}\beta_{2}^{4}+2\beta_{1}^{2}\beta_{2}^{2}d^{8}s-2\beta_{1}^{2}\beta_{2}^{2}d^{8}-18\beta_{1}^{2}\beta_{2}^{2}d^{6}s+18\beta_{1}^{2}\beta_{2}^{2}d^{6}+56\beta_{1}^{2}\beta_{2}^{2}d^{4}s-56\beta_{1}^{2}\beta_{2}^{2}d^{4}-72\beta_{1}^{2}\beta_{2}^{2}d^{2}s+72\beta_{1}^{2}\beta_{2}^{2}d^{2}+32\beta_{1}^{2}\beta_{2}^{2}s-32\beta_{1}^{2}\beta_{2}^{2}+\beta_{1}^{2}d^{8}s^{2}-2\beta_{1}^{2}d^{8}s+\beta_{1}^{2}d^{8}-12\beta_{1}^{2}d^{6}s^{2}+24\beta_{1}^{2}d^{6}s-12\beta_{1}^{2}d^{6}+52\beta_{1}^{2}d^{4}s^{2}-104\beta_{1}^{2}d^{4}s+52\beta_{1}^{2}d^{4}-96\beta_{1}^{2}d^{2}s^{2}+192\beta_{1}^{2}d^{2}s-96\beta_{1}^{2}d^{2}+64\beta_{1}^{2}s^{2}-128\beta_{1}^{2}s+64\beta_{1}^{2}+\beta_{2}^{4}d^{8}s-\beta_{2}^{4}d^{8}-8\beta_{2}^{4}d^{6}s+8\beta_{2}^{4}d^{6}+24\beta_{2}^{4}d^{4}s-24\beta_{2}^{4}d^{4}-32\beta_{2}^{4}d^{2}s+32\beta_{2}^{4}d^{2}+16\beta_{2}^{4}s-16\beta_{2}^{4}+2\beta_{2}^{2}d^{8}s^{2}-4\beta_{2}^{2}d^{8}s+2\beta_{2}^{2}d^{8}-24\beta_{2}^{2}d^{6}s^{2}+48\beta_{2}^{2}d^{6}s-24\beta_{2}^{2}d^{6}+104\beta_{2}^{2}d^{4}s^{2}-208\beta_{2}^{2}d^{4}s+104\beta_{2}^{2}d^{4}-192\beta_{2}^{2}d^{2}s^{2}+384\beta_{2}^{2}d^{2}s-192\beta_{2}^{2}d^{2}+128\beta_{2}^{2}s^{2}-256\beta_{2}^{2}s+128\beta_{2}^{2}+d^{8}s^{3}-3d^{8}s^{2}+3d^{8}s-d^{8}-16d^{6}s^{3}+48d^{6}s^{2}-48d^{6}s+16d^{6}+96d^{4}s^{3}-288d^{4}s^{2}+288d^{4}s-96d^{4}-256d^{2}s^{3}+768d^{2}s^{2}-768d^{2}s+256d^{2}+256s^{3}-768s^{2}+768s-256$.

Then $x_{2}^{*}$ can be obtained by Eq.5, which is:

$$x_{2}^{*}=\left\{ \frac{\beta_{2}\left( 2-d^{2} \right)\left[ \left( 2-d^{2} \right)\beta_{2}\theta\sqrt{x_{1}^{*}}+\left( 2+d \right)\left( a-c+cd \right)-d\beta_{1}\sqrt{x_{1}^{*}} \right]}{\left( 1-s \right)\left( d^{2}-4 \right)^{2}-\beta_{2}^{2}\left( 2-d^{2} \right)^{2}} \right\}^{2} .$$

Therefore, $p_{1}^{*} and p_{2}^{*}$ are respectively:

$$p_{1}^{*}=\frac{2a+2c+ad+cd-2\beta_{1}\sqrt{x_{1}^{*}}-\beta_{2}d\sqrt{x_{2}^{*}}-\beta_{2}d\theta\sqrt{x_{1}^{*}}}{4-d^{2}} ,$$

$$p_{2}^{*}=\frac{2a+2c+ad+cd-2\beta_{2}\sqrt{x_{2}^{*}}-\beta_{1}d\sqrt{x_{1}^{*}}-2\beta_{2}\theta\sqrt{x_{1}^{*}}}{4-d^{2}} .$$
